# Safety and efficacy of outpatient follow-up for referred patients with undiagnosed fever: A retrospective cohort study

**DOI:** 10.1097/MD.0000000000018532

**Published:** 2020-01-31

**Authors:** Yukinori Harada, Mikako Masuda, Takanobu Hirosawa, Hiroshi Takase, Kohei Morinaga, Michihiro Nin, Taro Shimizu

**Affiliations:** Dokkyo Medical University Hospital, Mibu-machi, Shimotsuga-gun, Tochigi, Japan.

**Keywords:** fever, outpatient, remission, undiagnosed

## Abstract

Fever is one of the most common symptoms seen in patients. The work-up and follow-up of fever in an outpatient-only setting is a reasonable option for stable patients referred for unexplained fever; however, the safety and efficacy of outpatient follow-up for those patients remain unclear. We conducted this study to evaluate the safety and efficacy of outpatient follow-up for referred patients with unexplained fever.

This study was a retrospective cohort study. We included patients referred to the outpatient department of the diagnostic medicine of our university hospital for unexplained fever between October 2016 and September 2017. Exclusion criteria were recurrent fever or admission for fever evaluation prior to referral. Main outcomes of interest were the rate of admission without diagnosis, rate of remission of fever, and the total duration of fever in undiagnosed patients.

Among 84 patients included in this study, 17 (20%) were diagnosed during outpatient follow-up, 6 (7%) were admitted due to worsened condition, 5 (6%) were lost to follow-up, and 56 (67%) were followed up as outpatients without a diagnosis. Among the 56 undiagnosed patients, fever resolved in 53 during outpatient follow-up with or without treatment (95%). The total duration of resolved fever in undiagnosed patients was within 8 weeks.

Follow-up of patients referred for unexplained fever in an outpatient setting is safe and effective.

## 1 Introduction

Fever is one of the most common symptoms seen in patients. The diagnostic strategy for patients with fever is well established, even in patients with fever of unknown origin (FUO).^[1,2]^ Furthermore, patients with fever that remains unexplained after appropriate work-up show good prognosis.^[1,3,4]^ Previous studies have mainly focused on unexplained fever that followed up in inpatient setting; however, work-up of fever in an outpatient-only setting should be considered as an option for stable patients in the current clinical practice reflecting the fact that hospital admission raises the risk for several adverse events and most diagnostic tests for fever do not require admission. Therefore, the present study evaluates the diagnostic efficacy and safety of management in referred outpatient visits for unexplained fever.

## 2 Methods

### 2.1 Study population and protocol

The present study population included patients referred to the outpatient department of the diagnostic medicine of our university hospital (from other outpatient departments or different clinics and hospitals) between October 2016 and September 2017 in order to evaluate the cause of fever, regardless of its grade. Exclusion criteria encompassed patients with recurrent fever or admitted patients for fever evaluation prior to the referral. Recurrent fever was defined as a cyclical fever with fever-free intervals of at least 2 weeks.^[5,6]^ The decision of admission was left to the discretion of the physician following the assessment of the severity of the condition and needs of the diagnostic tests. There was no standard protocol for work-up of unexplained fever; however, in general, antibiotics were not prescribed to patients without bacterial infection and were discontinued at the time of the referral visit when no bacterial infection was found. The study protocol was approved by the Independent Ethics Committee of the Dokkyo Medical University Hospital. As this was a retrospective cohort study, and the study data were anonymous, the requirement for informed consent was waived by the ethics committee.

### 2.2 Data collection

We retrospectively reviewed medical charts and extracted data of the participants regarding age, sex, referral origin, duration of fever at the time of the referral visit, numbers of visits to doctors prior to referral, laboratory analyses prior to referral, imaging studies prior to referral, bacterial culture analyses prior to referral, prescriptions of antibiotics, nonsteroidal anti-inflammatory drugs (NSAIDs) and acetaminophen prior to referral, admission after the referral visit without diagnosis, diagnosis, therapy, defervescence or not, and total duration of fever. The diagnoses recorded in medical charts were reviewed by the 2 authors (YH and MM) and confirmed based on the test results or clinical diagnostic criteria.

### 2.3 Outcomes

The outcomes of interest in this study were the ratio of admission for diagnosis (excluding patients admitted after diagnosis), ratio of patients followed up as outpatients without diagnosis, ratio of defervescence during follow-up in patients followed up as outpatients without diagnosis, and total duration of fever in patients followed up as outpatients without diagnosis.

### 2.4 Statistical analysis

Continuous data are presented as median (25th and 75th percentiles) due to non-normal distribution. Categorical data are presented as counts and proportions (%). All analyses were performed using the statistical software R, version 3.5.1 (Vienna, Austria).

## 3 Results

**Figure 1:**
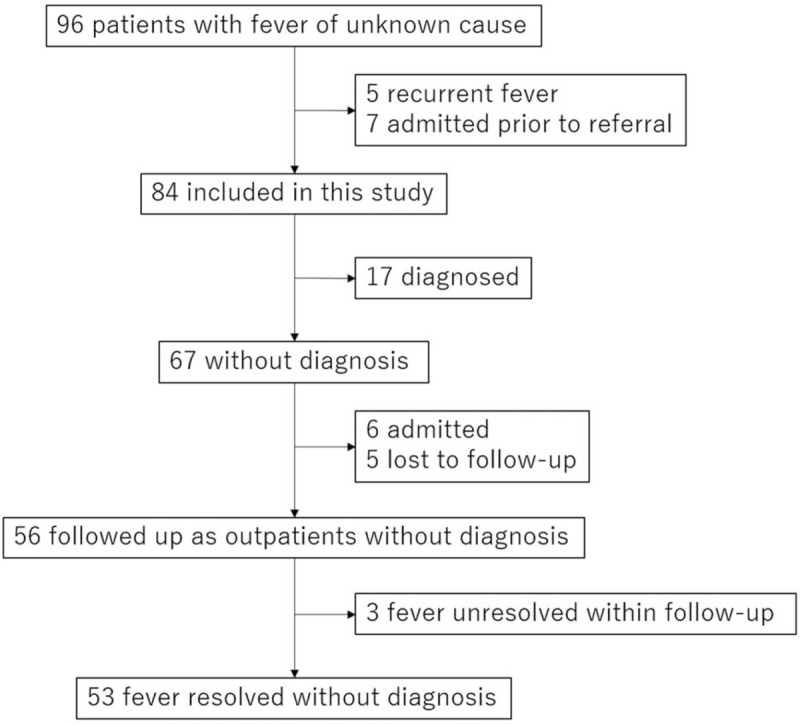
Flowchart of patient recruitment.

A flowchart showing the patient recruitment to the present study is shown in Fig. 1. A total of 84 patients were included. Patient characteristics at the time of the referral visit are shown in Table 1. Although 75% of patients were referred within 3 weeks after onset of fever, 50% visited other doctors ≥3 times, and several attempts to evaluate the cause of fever were made prior to referral. Antibiotics were prescribed prior to referral in around 50% of patients.

**Table 1:**
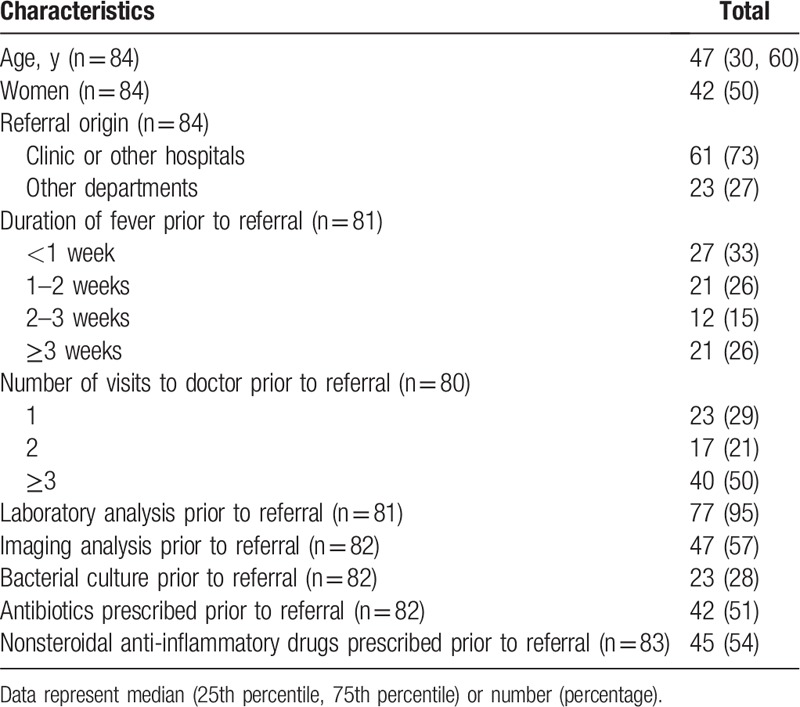
Baseline characteristics.

Seventeen (20%) patients were diagnosed during outpatient follow-up: 12 had infectious diseases, 3 had noninfectious inflammatory diseases, and 2 were classed as miscellaneous. In the remaining patients without a diagnosis, 6 (7%) were admitted due to worsened condition and 5 (6%) lost to follow-up. A total of 56 patients (67%) were followed up as outpatients without a diagnosis. Among them, none died, and fever resolved in 53 during follow-up with or without treatment (95%).

**Figure 2:**
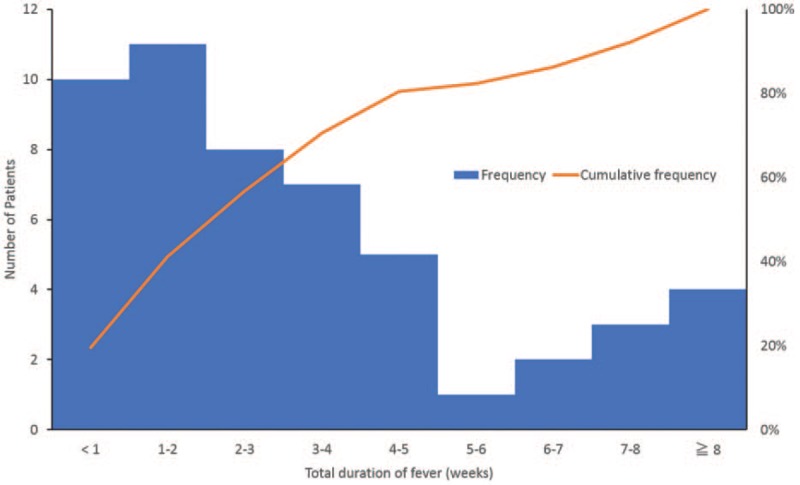
Total duration of fever in patients whose fever resolved during outpatient follow-up without diagnosis. Blue columns indicate a histogram of the total duration of fever, and height represents the number of patients. The orange line represents the cumulative percentage of patients.

Figure 2 shows the total duration of fever in 53 undiagnosed patients whose fever resolved during outpatient follow-up with or without treatment. Fever sustained for 3 weeks the least in 63% of patients, but almost all reported defervescence within 8 weeks. These patients were either not treated (n = 17; 32%) or treated using acetaminophen or NSAIDs (n = 32; 60%), antibiotics (n = 3; 6%), or Chinese herbal medicine (n = 1; 2%).

## 4 Discussion

The present study found that only 7% of referred outpatients required admission without a diagnosis. While the diagnostic rate was low (20%), almost all undiagnosed patients recovered within <8 weeks with or without treatment.

Admission solely to detect the cause of fever may be avoided in most cases. The admission rate of FUO cases decreased from 89% in 1990 to 1999 to 68% in 2005 to 2014.^[3,6]^ The extremely low proportion (7%) of admission in the present study may be attributed to several factors, including hospital ward capacity and preference for outpatient care by both the physicians and patients based on the high costs and inconvenience incurred by hospitalization. In addition, the high ratio (67%) of undiagnosed patients in the present study may be associated with low admission rate. A recent study showed a lower admission rate in patients with undiagnosed FUO compared with diagnosed FUO.^[3]^ Previous studies also indicated that undiagnosed patients showed fewer symptoms or abnormal findings compared with the diagnosed patients.^[2,7]^ Thus, it is assumed that undiagnosed patients tend to be less ill and show less diagnostic evidence requiring further in-depth tests.

Regarding the low rate of diagnosed patients in the present study, lack of standard protocol for fever work-up may be the primary cause. Recent prospective cohort studies using a standardized protocol showed a reduced rate of undiagnosed patients under 20% in all except for 1 study (51%).^[2,8–10]^ Our study did not include some of the tests as routine work-up included in these studies, such as cytomegalovirus serology test, while cytomegalovirus infection was reported to be a relatively common cause of FUO.^[2,8–11]^ We did not use these tests routinely because we thought the results of these tests were not associated with the prognosis and therefore were not cost-effective in the management; however, we may have missed some diagnosable diseases. Furthermore, as we included patients whose fever persisted within 3 weeks in the present study, it was assumed that these patients suffered from a self-limiting viral infection that was undetectable by routine analyses and led to a decrease in the diagnostic rate. This hypothesis was supported by our findings of higher remission rate (95%) and shorter duration (within 8 weeks) of fever in undiagnosed patients compared with previous studies (remission rate, 43.8%–91.8%; duration, >3 months),^[2,3,12–14]^ and was also supported by the reported data of high remission rate (96%) in a study that included FUO patients with a relatively shorter duration of fever (mean, 26 days).^[15]^ Therefore, our study demonstrated that undiagnosed patients with a relatively short duration of fever resolved within 8 weeks.

Our study results also suggest that empirical therapy with antibiotics could be unnecessary and supportive therapy with acetaminophen or NSAIDs are enough for clinically stable outpatients with a short duration of unexplained fever. Of note, in our study, among patients whose fever resolved without a definitive diagnosis, 32% did not need any treatment, and 60% only needs acetaminophen or NSAIDs, and only 6% required antibiotics. Our results were consistent with the data from an observational cohort study that remission of fever occurred in around half of the untreated patients with undiagnosed FUO.^[16]^ Our results were also consistent with the data that the remission rate of fever was comparable between who treated with NSAIDs or acetaminophen and antibiotics among patients with undiagnosed FUO.^[16]^ These data support the recommendation not to use empirical antibiotics and the recommendation from experts to use NSAIDs for treatment to patients with undiagnosed fever.^[17,18]^ Positron emission tomography–computed tomography (PET-CT) can be helpful to reduce overtreatment for patients with undiagnosed fever with its predictive value for spontaneous remission.^[19]^ However, because we did not use PET-CT in almost all patients in this study, the usefulness of PET-CT for undiagnosed patients with a relatively short duration of fever is still unknown.

The present study has 2 limitations. First, our results may have been biased and included missing data due to its retrospective nature and a lack of protocol for fever work-up. Second, our results cannot be easily generalized since the study was conducted only in one hospital. On the other hand, the participants included in our study were more similar to the patients who usually present to the outpatient department of general internal medicine than those who were included in other FUO studies focusing on inpatient setting. Furthermore, the routine tests performed in our study can be carried out in community hospitals. Therefore, our results are valuable for clinicians who usually diagnose patients with fever.

## 5 Conclusions

Follow-up of patients referred for unexplained fever in outpatient setting may be a safe and effective option.

## Acknowledgments

The authors would like to thank Enago (www.enago.jp) for the English language review.

## Author contributions

**Conceptualization:** Yukinori Harada, Taro Shimizu.

**Data curation:** Yukinori Harada, Mikako Masuda, Hiroshi Takase.

**Formal analysis:** Yukinori Harada.

**Investigation:** Yukinori Harada, Mikako Masuda, Takanobu Hirosawa, Hiroshi Takase.

**Methodology:** Yukinori Harada, Takanobu Hirosawa.

**Project administration:** Yukinori Harada, Taro Shimizu.

**Resources:** Yukinori Harada, Mikako Masuda, Michihiro Nin.

**Supervision:** Taro Shimizu.

**Writing – original draft:** Yukinori Harada.

**Writing - review & editing:** Mikako Masuda, Takanobu Hirosawa, Hiroshi Takase, Kohei Morinaga, Michihiro Nin, Taro Shimizu.

Yukinori Harada orcid: 0000-0001-6042-7397

Mikako Masuda orcid:0000-0003-2924-1572

Takanobu Hirosawa orcid:0000-0002-3573-8203

Hiroshi Takase orcid: 0000-0001-7339-4109

Kohei Morinaga orcid: 0000-0003-4811-4085

Michihiro Nin orcid: 0000-0001-7889-2617

Taro Shimizu orcid:0000-0002-3788-487X
